# Critical power is a key threshold determining the magnitude of post‐exercise hypotension in non‐hypertensive young males

**DOI:** 10.1113/EP091429

**Published:** 2023-09-15

**Authors:** Tze‐Huan Lei, I‐Lin Wang, Yi‐Ming Chen, Xin‐Hao Liu, Naoto Fujii, Shunsaku Koga, Blake Perry, Toby Mundel, Faming Wang, Yinhang Cao, Kohei Dobashi, Narihiko Kondo, Hao‐Yu Li, Richie P. Goulding

**Affiliations:** ^1^ College of Physical Education Hubei Normal University Huangshi China; ^2^ Department of Food Science Fu Jen Catholic University New Taipei City Taiwan; ^3^ Faculty of Health and Sport Sciences University of Tsukuba Tsukuba Japan; ^4^ Applied Physiology Laboratory Kobe Design University Kobe Japan; ^5^ School of Health Sciences Massey University Wellington New Zealand; ^6^ Department of Kinesiology Brock University St Catharines Canada; ^7^ Division Animal and Human Health Engineering, Department of Biosystems (BIOSYST) KU Leuven Leuven Belgium; ^8^ School of Athletic Performance Shanghai Sport University Shanghai China; ^9^ Faculty of Education Hokkaido University of Education Asahikawa Japan; ^10^ Laboratory for Applied Human Physiology, Graduate School of Human Development and Environment Kobe University Kobe Japan; ^11^ Laboratory for Myology, Department of Human Movement Sciences, Faculty of Behavioral and Human Movement Sciences, Vrije Universiteit Amsterdam Amsterdam Movement Sciences Amsterdam the Netherlands

**Keywords:** cardiovascular health, critical power, exercise intensity, post‐exercise baroreflex

## Abstract

The effect of different exercise intensities on the magnitude of post‐exercise hypotension has not been rigorously clarified with respect to the metabolic thresholds that partition discrete exercise intensity domains (i.e., critical power and the gas exchange threshold (GET)). We hypothesized that the magnitude of post‐exercise hypotension would be greater following isocaloric exercise performed above versus below critical power. Twelve non‐hypertensive men completed a ramp incremental exercise test to determine maximal oxygen uptake and the GET, followed by five exhaustive constant load trials to determine critical power and *W′ (work available above critical power)*. Subsequently, criterion trials were performed at four discrete intensities matched for total work performed (i.e., isocaloric) to determine the impact of exercise intensity on post‐exercise hypotension: 10% above critical power (10% > CP), 10% below critical power (10% < CP), 10% above GET (10% > GET) and 10% below GET (10% < GET). The post‐exercise decrease (i.e., the minimum post‐exercise values) in mean arterial (10% > CP: −12.7 ± 8.3 vs. 10% < CP: v3.5 ± 2.9 mmHg), diastolic (10% > CP: −9.6 ± 9.8 vs. 10% < CP: −1.4 ± 5.0 mmHg) and systolic (10% > CP: −23.8 ± 7.0 vs. 10% < CP: −9.9 ± 4.3 mmHg) blood pressures were greater following exercise performed 10% > CP compared to all other trials (all *P* < 0.01). No effects of exercise intensity on the magnitude of post‐exercise hypotension were observed during exercise performed below critical power (all *P* > 0.05). Critical power represents a threshold above which the magnitude of post‐exercise hypotension is greatly augmented.

## Abstract (highlights)

- **What is the central questions of this study?**
  What is the influence of exercise intensity on the magnitude of post‐exercise hypotension with respect to metabolic thresholds?
- **What is the main finding and its importance?**
  The magnitude of post‐exercise hypotension is greatly increased following exercise performed above critical power. However, below critical power, there was no clear effect of exercise intensity on the magnitude of post‐exercise hypotension.

## 1 INTRODUCTION

The benefits of exercise with regard to cardiovascular risk are widely appreciated (Myers et al., 2004, 2002; Sharman et al., 2015), and therefore establishing the mechanisms by which exercise reduces cardiovascular risk is of paramount importance. One such mechanism is via a reduction in blood pressure (Sharman et al., 2015; Tipton, 1984). Over time, chronic endurance exercise training can decrease resting blood pressure (Tipton, 1984). Moreover, mean arterial pressure (MAP) can be transiently decreased following an acute bout of exercise, termed ‘post‐exercise hypotension’ (Fitzgerald, 1981; MacDonald, 2002; MacDonald et al., 1999). This effect can persist for 1–24 h, and it has been suggested that repeated instances of post‐exercise hypotension are mechanistically linked to the chronic reduction in systolic blood pressure following endurance training (MacDonald, 2002). Establishing the characteristics of an exercise bout that are necessary to elicit post‐exercise hypotension is therefore essential for the design of effective interventions aimed at reducing blood pressure, and more broadly, for understanding the benefits of exercise for the cardiovascular system.

One such variable that has the potential to impact the presence and magnitude of post‐exercise hypotension is exercise intensity. Despite this, many studies have failed to find an effect of exercise intensity on the magnitude or occurrence of post‐exercise hypotension (Jones et al., 2007, 2021; MacDonald et al., 1999). This has led some to suggest that post‐exercise hypotension is more strongly mediated by total work done, rather than exercise intensity (Jones et al., 2007). It is worth noting that most studies examining the influence of exercise intensity on post‐exercise hypotension have normalized exercise intensity as a percentage of the maximal ${\dot{V}}_{O_{2}}$ (${\dot{V}}_{O_{2}\max}$) (Jones et al., 2007; MacDonald et al., 1999) or maximal heart rate (Jones et al., 2021; Mündel et al., 2015). Both of these methods fail to account for the thresholds separating intensity domains (i.e., lactate threshold, or its non‐invasive surrogate the gas exchange threshold (GET) and critical power), increasing the likelihood that not all participants were exercising within the same intensity domains (Iannetta et al., 2020, 2021). For instance, during exercise below the GET (i.e. moderate exercise), pulmonary and muscle oxygen uptake (${\dot{V}}_{O_{2}}$) reach a steady‐state in 2–3 min in most healthy individuals and blood [lactate] is stable across time (Black et al., 2017; Grassi et al., 1996). Above the GET but below critical power (i.e. heavy exercise), the attainment of a ${\dot{V}}_{O_{2}}$ steady‐state is delayed by up to 20 min and blood [lactate] is elevated above resting values but stable with time (Jones et al., 2011; Poole et al., 1994; Whipp, 1994). Above critical power (i.e. severe exercise), a steady‐state is unattainable for both ${\dot{V}}_{O_{2}}$ and blood [lactate], with ${\dot{V}}_{O_{2}\max}$ being attained shortly prior to task failure (Goulding & Marwood, 2023; Goulding et al., 2021; Jones et al., 2011; Poole et al., 2016). In practical terms, healthy individuals perform most activities of daily living below GET, whereas for those with chronic diseases, the elderly and very inactive individuals, activities of daily living may be carried out above GET and/or critical power (Goulding & Marwood, 2023; Poole et al., 2021). These thresholds therefore partition the full range of aerobic activities performed in daily life.

As the physiological responses to exercise differ so markedly between each domain, there is the possibility that the failure of previous studies to find an effect of exercise intensity on the magnitude of post‐exercise hypotension was due to those studies not appropriately accounting for metabolic thresholds, which occur at highly variable fractions of ${\dot{V}}_{O_{2}\max}$ between individuals (Iannetta et al. (2020, 2021); recently highlighted in this journal by Meyler et al. (2023) and Poole and Jones (2023)). Exercise performed above critical power results in a greater post‐exercise increase in vascular conductance when compared to sub‐critical power exercise (Hammer et al., 2021), and post‐exercise changes in vascular conductance are closely linked to the occurrence of post‐exercise hypotension (Halliwill et al., 2000, 1996). This suggests that supra‐critical power exercise might represent a more effective stimulus to elicit post‐exercise hypotension and/or increase its magnitude. A comparison of the magnitude of post‐exercise hypotension following exercise above versus below critical power would be required to test this hypothesis. However, a rigorous analysis of the magnitude of post‐exercise hypotension according to the intensity domain schema (Whipp, 1996) has not yet been performed. If critical power can be demonstrated to represent a threshold intensity above which a greater magnitude of post‐exercise hypotension may be elicited in healthy non‐hypertensive individuals, this would provide a strong rationale for employing supra‐critical power exercise as a strategy to reduce resting blood pressure in hypertensive individuals in future research. Moreover, resolving the effect of exercise intensity on post‐exercise hypotension is an important issue to understand for the prevention of post‐exercise syncope (Halliwill et al., 2014).

The purpose of this study was to examine the effect of exercise intensity on the magnitude of post‐exercise hypotension. We hypothesized that the magnitude of post‐exercise hypotension would not be different for moderate versus heavy exercise, but greater for severe exercise. Evidence in support of this hypothesis would implicate critical power as a key threshold for determining the magnitude of post‐exercise hypotension.

## 2 METHODS

### 2.1 Participants

An a priori power analysis (G*Power version 3.1.9.4; Heinrich Heine University Düsseldorf, Düsseldorf, Germany) showed that a minimum of eight participants was required based on conventional α (0.05) and β (0.80) values, and an effect size of 0.51 as reported by Jones et al. (2007) using the post‐exercise reduction in MAP as the primary dependent variable. Therefore, twelve healthy, recreationally active but untrained, non‐hypertensive (Table 1) Asian men were recruited for this study. Although recruitment was intended to target both males and females, the three females who initially took part in the study dropped out during the critical power determination trials prior to the criterion trials. This study was approved by the University Joint Institute Review Board (JLSU‐IRB2022001) and performed in accordance with the latest version of the *Declaration of Helsinki* and registration with the China Clinical database (ChiCTR2300071853). Each participant provided verbal and written informed consent prior to participation.

**TABLE 1:** Subject characteristics.

Subject	Age (yr)	Systolic BP (mmHg)	Diastolic BP (mmHg)	${\dot{V}}_{O_{2}\max}$ (L.min^−1^)	Peak power output (watts)
1	25	127	80	3.90	338
2	25	119	79	3.30	300
3	25	125	77	2.50	255
4	23	138	81	3.55	245
5	24	135	79	3.0	275
6	24	115	79	3.50	279
7	24	110	77	3.14	254
8	24	130	86	2.60	213
9	22	126	76	3.30	295
10	24	116	82	3.70	286
11	24	135	85	2.20	215
12	24	114	79	2.13	227
Mean	24	124	80.0	3.10	265
SD	0.90	9	3.1	0.6	37

### 2.2 Experimental overview

All participants reported to a temperature‐controlled laboratory (24.5 ± 0.5°C, 50.5 ± 5% relative humidity) on 10 separate occasions: (1): a maximal ramp incremental exercise test, (2) five critical power determination trials and (3) four experimental trials where the occurrence and magnitude of post‐exercise hypotension were monitored during exercise and recovery from bouts at four intensities: 10% above critical power (i.e., 10% > CP; severe), 10% below critical power (i.e., 10% < CP; heavy), 10% above the gas exchange threshold (i.e., 10% > GET; heavy) and 10% below the GET (i.e., 10% < GET; moderate). Each trial was separated by at least 48 h to ensure adequate recovery. Each experimental trial was conducted at the same time of day to eliminate the effect of diurnal rhythm on blood pressure and body temperature fluctuations and performed 2 h postprandial. Furthermore, participants abstained from alcohol/caffeine intake and avoided high‐sodium foods 48 h prior to all experimental trials. Following the critical power determination trials, participants first completed the 10% > CP trial (performed until task failure) in order to facilitate comparisons at subsequent intensities matched for total work done in the 10% > CP trial. The subsequent trials were performed in a randomized order.

### 2.3 Preliminary testing and the critical power trials

All participants completed a maximal ramp incremental exercise test on a cycle ergometer (Ergoline, Bitz, Germany) for determination of ${\dot{V}}_{O_{2}\max}$, the GET (a non‐invasive surrogate for the lactate threshold), and the power outputs for subsequent visits. Each test consisted of a 4‐min baseline period of cycling at 20 W, followed by a ramped, linear increase in work rate of 20 W/min at a fixed cadence of 70 rpm until the participant could no longer maintain the cadence above 60 rpm despite strong verbal encouragement. Ventilatory and gas exchange variables were measured continuously breath‐by‐breath throughout each test. ${\dot{V}}_{O_{2}\max}$ was confirmed by either (1) the occurrence of a plateau in ${\dot{V}}_{O_{2}}$ despite an increasing workload during the ramp incremental test or (2) the occurrence of a plateau in the plot of ${\dot{V}}_{O_{2}}$ versus power output when determined from the discontinuous tests used to determine critical power, as described by Day et al. (2003). The GET was independently verified by two different investigators (T.H.L. and R.P.G.) using the following criteria: (1) a disproportionate increase in CO_2_ production (${\dot{V}}_{{\mathrm{CO}}_{2}}$) relative to ${\dot{V}}_{O_{2}}$; (2) an increase in the ventilatory equivalent for O_2_ (i.e., ${\dot{V}}_{E}$/${\dot{V}}_{O_{2}}$) without an increase in the ventilatory equivalent for CO_2_ (i.e., ${\dot{V}}_{E}$/${\dot{V}}_{{\mathrm{CO}}_{2}}$); and (3) an increase in end tidal O_2_ tension with no change in end tidal CO_2_ tension. All power outputs utilized in subsequent tests were corrected by the ${\dot{V}}_{O_{2}}$ mean response time, which was determined as described previously (Goulding et al., 2017, 2020).

The power–duration relationship was determined by five constant power output trials that were selected to elicit a range of times to task failure spanning 2–15 min. These power outputs were initially selected based on performance in the incremental test and calculated to be in the range of 50%Δ (i.e., a work rate calculated to require 50% of the difference between GET and ${\dot{V}}_{O_{2}\max}$) to 110% ${\dot{V}}_{O_{2}\max}$. All tests began with a 4 min period of baseline pedalling at 20 W, before an abrupt transition to the desired power output was applied and participants exercised until task failure. Time to task failure was recorded to the nearest second. If the peak ${\dot{V}}_{O_{2}}$ attained at the end of any given trial was less than 95% of that attained in the incremental test, that trial was repeated. Conversely, if the peak ${\dot{V}}_{O_{2}}$ attained at the end of any given trial was greater than 105% of that attained in the incremental test, the incremental test was repeated. In all cases, ${\dot{V}}_{O_{2}\max}$ was verified.

### 2.4 Post‐exercise hypotension trials

The four different exercise intensities employed in the post‐exercise hypotension trials were power outputs selected to elicit physiological responses consistent with moderate, heavy and severe intensity exercise. These intensities were 10% < GET (moderate), 10% > GET (heavy), 10% < CP (heavy) and 10% > CP (severe). The duration of all sub‐critical power trials was modified to ensure that the total work done (isocaloric) in each trial was the same, allowing comparison of the effect of exercise intensity, independent from work done, on post‐exercise hypotension. Following each trial, the gas exchange and ventilatory responses were inspected to ensure that they reflected the intended exercise intensity domain. If this was not the case, the intensity was modified and the trial was repeated on a separate day. Following each exercise bout, participants recovered whilst seated on the cycle ergometer for 60 min, during which time their physiological responses were continuously recorded.

### 2.5 Measurements

#### 2.5.1 Anthropometric

Height and body mass were measured using a stadiometer (Seca, Hamburg, Germany; accurate to 0.1 cm) and scale (Jadever, Taipei, Taiwan; accurate to 10 g), from which body surface area was estimated.

#### 2.5.2 Respiratory

Expired respiratory gases were collected from a mixing chamber and analysed for ${\dot{V}}_{O_{2}}$, ${\dot{V}}_{{\mathrm{CO}}_{2}}$ and ${\dot{V}}_{E}$ using an online, breath‐by‐breath system (Max II, AEI Technologies, Bastrop, TX, USA). Data were recorded breath‐by‐breath and smoothed using a 10 s average during the ramp incremental test and every 3 s during the subsequent trials. This system was calibrated before each trial using zero and β‐standard gas concentrations and a 3‐litre syringe (Hans Rudolph (Shawnee, KS, USA) 3L Calibration Syringe), according to the manufacturer's instructions.

#### 2.5.3 Cardiovascular

Heart rate was continuously recorded from the detection of R–R intervals (Polar Vantage XL, Polar Electro, Kempele, Finland) while brachial artery blood pressure was measured manually via sphygmomanometry. Blood pressure measurements were performed in duplicate by the same experienced operator at rest, end‐exercise and every 5 min during the recovery period. MAP was calculated as diastolic blood pressure + 1/3 pulse pressure. Vascular stiffness was estimated via brachial ankle pulse wave velocity (baPWV; BP‐203RPE III, Omron, Kyoto, Japan) before and after 60 min of the recovery as a surrogate of arterial compliance (Kingwell et al., 1997).

#### 2.5.4 Body temperatures

As core temperature (*T*_core_) can modulate changes in post‐exercise vascular compliance, which could in turn influence the magnitude of post‐exercise hypotension, we measured *T*_core_ to provide insight into this potential mechanism. *T*_core_ was measured by a rectal thermistor (TMQ‐DAG, Unimed, Beijing, China; accurate to 0.1°C). Mean skin temperature (*T*_sk_) was measured at four sites using calibrated skin thermistors (Grant Instruments (Cambridge) Ltd, Royston, UK; accurate to 0.2°C) fastened on the calf, thigh, chest and forearm using surgical tape (3M Healthcare, Maplewood, MN, USA). Regional‐weighted mean *T*_sk_ was calculated according to the equation of Ramanathan (1964).

#### 2.5.5 Haematological variables

In each session before, immediately after exercise and following 1 h recovery, capillary blood samples were taken from the fingertip in duplicate. Whole blood was used to measure haemoglobin concentration (Hemo Control, EKF diagnostics, Barleben, Germany). Subsequently, haematocrit was calculated and the plasma volume changes were calculated via the Dill and Costill equation (Dill & Costill, 1974).

### 2.6 Data analysis

Critical power and *W′* (work capacity available above critical power) were estimated using the three following models: the hyperbolic power–time model where time to task failure is plotted against power output (Equation 1); the linear work‐time model where time is plotted against work done (Equation 2) and the linear inverse of time model where power is plotted against the inverse of time (Equation 3), as follows:

(1) $$P\;=\frac{W^{\prime }}{T}\;+\mathrm{CP}\;$$

(2) $$W\;=\;\mathrm{CP}\times T+W^{\prime }$$

(3) $$P\;=W^{\prime }\;\times \left(1/T\right)+\mathrm{CP}$$

Where *P* is power, *T* is time, *W* is work done, CP is critical power and *W′* is the finite work capacity available above critical power. The standard error of the parameter estimates for critical power and *W′* were expressed as a coefficient of variation relative to the parameter estimate (CV%). The model with the lowest average CV% was then used for all subsequent analyses.

#### 2.6.1 Statistical analysis

As post‐exercise hypotension is defined as the post‐exercise change in MAP relative to the baseline value, systolic and diastolic blood pressure and MAP were analysed and presented as the change from the baseline value. For clarity, absolute data are also presented. Due to the large number of levels for both exercise intensity and recovery time factors, the post‐exercise minimum values in each of these variables were also analysed and are presented as summary statistics. All statistical analyses were performed with SPSS software for Windows (IBM SPSS Statistics 20, IBM Corp., Armonk, NY, USA) and figures were produced using GraphPad Prism (version 7.00, GraphPad Software, San Diego, CA, USA). Homogeneity of variance was examined by Levene's test, and the normality of the data was examined by the Kolmogorov–Smirnov test. To investigate the effect of exercise intensity on the magnitude of post‐exercise hypotension, the post‐exercise minimum values for systolic blood pressure, diastolic blood pressure and MAP were analysed by one‐way repeated ANOVA. Values for ${\dot{V}}_{O_{2}}$ at baseline and end‐exercise across the different conditions were also compared by one‐way repeated measures ANOVA, whereas end‐exercise ${\dot{V}}_{O_{2}}$ for the 10% > CP trial was compared with that attained during the ramp incremental test via paired *t*‐test. The remaining physiological variables were analysed using two‐way repeated (time × intensity) ANOVA. In all cases, where main or interaction effects occurred, *post hoc* pairwise analyses were performed using the Holm–Bonferroni correction method. Data are presented are means ± SD, unless stated otherwise. Statistical significance was accepted at *P* < 0.05.

## 3 RESULTS

### 3.1 Incremental test and power‐duration relationship

${\dot{V}}_{O_{2}\max}$ was 3.10 ± 0.6 l/min (40.8 ± 8.1 ml/kg/min) and the GET occurred at 1.8 ± 0.3 l/min (24.2 ± 4.4 ml/kg/min), with an associated power output of 119 ± 18 W. The model producing the best‐fit power–duration parameter estimates yielded a critical power of 151 ± 18 W (1.8 ± 0.9 CV%) and a *W′* of 19.1 ± 6.9 kJ (8.3 ± 5.5 CV%). Power–duration parameter estimates for each subject, along with the model used, are presented in Table 2.

**TABLE 2:** Individual best‐fit power–duration relationship parameters.

Subject	Best‐fit model	CP (W)	SEE (W)	CV (%)	*W′* (kJ)	SEE (kJ)	CV (%)
1	Hyperbolic	189	1.6	1	23.0	1.5	7
2	Hyperbolic	157	2.8	2	23.0	1.0	4
3	Hyperbolic	152	2.2	1	13.2	1.3	10
4	1/time	150	3.2	2	22.9	1.1	5
5	Work–time	150	6.4	4	14.3	3.3	23
6	Work–time	173	1.2	1	17.6	0.5	3
7	Hyperbolic	144	2.6	2	16.0	1.8	11
8	1/time	121	4.2	3	15.6	1.2	8
9	Hyperbolic	141	2.8	2	29.9	1.7	6
10	Hyperbolic	157	2.6	2	31.4	1.7	5
11	Hyperbolic	128	1.7	1	10.5	1.3	13
12	Hyperbolic	144	0.9	1	11.9	0.6	5
Mean		150.5	2.7	1.8	19.1	1.4	8.3
SD		18.2	1.5	0.9	6.9	0.7	5.5

Abbreviations: CP, critical power; CV, coefficient of variation; SEE, standard error of the estimate; *W′*, work available above critical power.

### 3.2 Exercise intensity estimation

**FIGURE 1:**
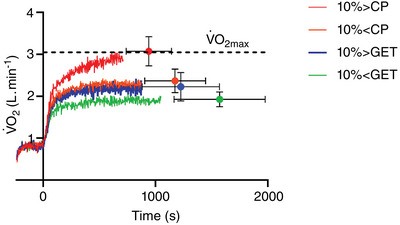
Group mean oxygen uptake (${\dot{V}}_{O_{2}}$) during the criterion trials used to determine the effect of exercise intensity on the magnitude of post‐exercise hypotension. Red indicates severe exercise performed 10% above critical power (CP), orange indicates heavy exercise performed 10% below CP, blue indicates heavy exercise performed 10% above the gas exchange threshold (GET), and green indicates moderate exercise performed 10% below the GET. Horizontal dashed line indicates the maximal ${\dot{V}}_{O_{2}}$ determined from the ramp incremental test. The final data point in each condition represents the group mean ± SD ${\dot{V}}_{O_{2}}$ during the final 20 s of each trial. The peak ${\dot{V}}_{O_{2}}$ attained at end‐exercise during the supra‐CP trial did not differ from the ${\dot{V}}_{O_{2}\max}$ determined during the ramp incremental test (*P* > 0.05).

The group mean ± SD ${\dot{V}}_{O_{2}}$ responses to exercise at each intensity employed for the experimental trials in shown in Figure 1. The 10% > CP trial was conducted at 166 ± 20 W. Task failure occurred within 944 ± 204 s (total work done = 157.6 ± 44.4 kJ) and the ${\dot{V}}_{O_{2}}$ attained at task failure did not differ from that attained during the incremental test (3.07 ± 0.34 l/min, *P* = 0.90), consistent with severe‐intensity exercise. The 10% < CP trial was conducted at 135 ± 16 W and lasted for 1161 ± 262 s (total work done = 157.7 ± 44.5 kJ). In all cases, the ${\dot{V}}_{O_{2}}$ attained at end‐exercise was below ${\dot{V}}_{O_{2}\max}$ (2.37 ± 0.28 l/min, 76 ± 13% ${\dot{V}}_{O_{2}\max}$, *P* < 0.01), yet greater than the value at 3 min (+0.25 ± 0.26 l/min, 68 ± 11% ${\dot{V}}_{O_{2}\max}$, *P* = 0.007), confirming that this was heavy‐intensity exercise. The 10% > GET trial was conducted at 130 ± 18 W (1224 ± 342 s, 157.5 ± 44.4 kJ), with heavy exercise confirmed by an increase in ${\dot{V}}_{O_{2}}$ from 3 min to end‐exercise (3 min: 2.01 ± 0.26 l/min, 65 ± 12% ${\dot{V}}_{O_{2}\max}$; end‐exercise: 2.23 ± 0.31 l/min, 72 ± 15% ${\dot{V}}_{O_{2}\max}$, *P* = 0.012). The 10% < GET trial was conducted at 106 ± 15 W (1495 ± 420 s, 157.6 ± 44.4 kJ). A steady‐state ${\dot{V}}_{O_{2}}$ indicative of moderate exercise was confirmed in all cases, with no difference in ${\dot{V}}_{O_{2}}$ between 3 min and end‐exercise (3 min: 1.78 ± 0.32 l/min, 57 ± 14% ${\dot{V}}_{O_{2}\max}$; end‐exercise: 1.86 ± 0.24 l/min, 60 ± 12% ${\dot{V}}_{O_{2}\max}$, *P* = 0.18).

### 3.3 Effect of exercise intensity on post‐exercise hypotension

**FIGURE 2:**
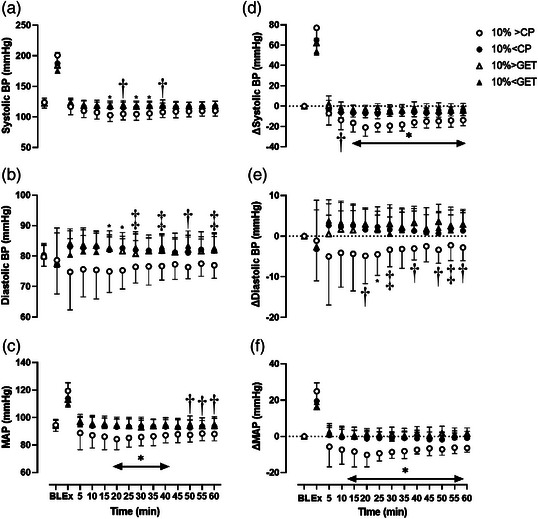
The group mean ± SD systolic blood pressure (SBP, a, d), diastolic blood pressure (DBP, b, e) and mean arterial pressure (MAP, c, f) at baseline (BL), end‐exercise (Ex) and throughout 60 min of recovery. Absolute values are displayed in (a–c), and relative changes from the resting baseline are displayed in panels (d–f). *10% > CP significantly different from all other trials; †10% > CP significantly different from 10% > GET and 10% < GET; ‡10% > CP significantly different from 10% < GET (*P* < 0.05).

**FIGURE 3:**
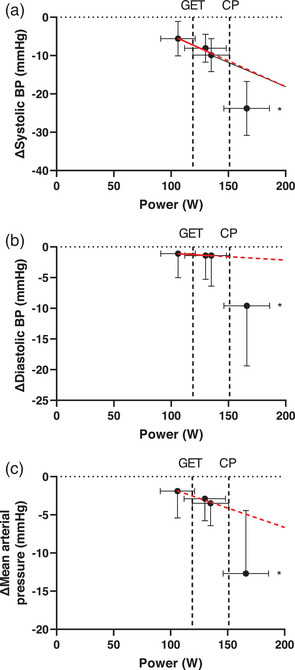
The effect of exercise intensity on the magnitude of post‐exercise hypotension. Values displayed are group mean ± SD values describing the greatest magnitude of post‐exercise hypotension to exercise at each of the four intensities comprising the criterion trials (i.e., 10% above critical power (CP), 10% below CP, 10% above the gas exchange threshold (GET) and 10% below GET). Systolic blood pressure is displayed in (a), diastolic blood pressure in (b) and mean arterial pressure in (c). Horizontal dashed lines reflect the group mean gas exchange threshold (GET) and critical power (CP). *10% > CP was significantly different from all other trials (all *P* < 0.05).

Resting systolic blood pressure, diastolic blood pressure and MAP were not different across the different exercise intensity trials (all *P* > 0.90). The post‐exercise reduction in systolic blood pressure was greater at 10% > CP when compared to all other intensities from 15 min until the end of recovery (Figure 2, *P* < 0.01). Furthermore, the reduction in systolic blood pressure was greater during recovery from 10% < CP exercise when compared to 10% < GET at 20, 25, 50 and 60 min (Figure 2). The post‐exercise reduction in diastolic blood pressure was greater during recovery from 10% > CP exercise between 15 and 30 min when compared to all other intensities (Figure 2, *P* < 0.01). Consequently, the post‐exercise reduction in MAP in the 10% > CP trial was greater than all other trials from 15 min until the end of the recovery period (Figure 2, *P* < 0.01). Similarly, the minimum post‐exercise values (i.e., as a change from baseline) for systolic blood pressure and MAP, reflecting the greatest magnitude of hypotension within the post‐exercise period, were lower after exercise at 10% > CP when compared to all other trials (Figure 3, *P* < 0.01), whereas for diastolic blood pressure the 10% > CP trial was lower compared to 10% < CP and 10% < GET only (Figure 3, all *P* < 0.05). The timing of the nadir values did not differ between intensities (MAP: 10% > CP, 28 ± 13 min; 10% < CP, 35 ± 18 min; 10% > GET, 27 ± 15 min; 10% < GET, 27 ± 15 min; *P* > 0.05).

### 3.4 Body temperature

**FIGURE 4:**
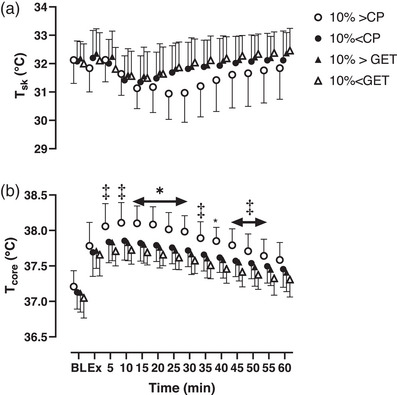
The effect of exercise intensity on the group mean ± SD core temperature response at rest, during exercise and following 1 h recovery. Open circles represent the exercise trial performed 10% above critical power (CP); filled circles represent the trial performed 10% below CP; filled triangles represent 10% above the gas exchange threshold (GET); open triangles represent the trial performed 10% below GET. *10% > CP was significantly different from all other trials (10% < CP, 10% > GET and 10% < GET); ‡10% > CP significantly different from 10% < GET (all *P* < 0.05).

Core temperature at rest was not different across trials (*P* = 0.90). Core temperature was greater during recovery from 15 to 30 min in the 10% > CP trial when compared to all other trials (*P* < 0.01, Figure 4). Furthermore, core temperature did not differ for all sub‐critical power trials throughout recovery (all *P* > 0.05, Figure 4). Skin temperature at rest was not different across trials (*P* = 0.90). There was an interaction effect between intensity and time on skin temperature (*P* < 0.01); however, post‐hoc tests failed to reveal any differences between trials (all *P* > 0.05).

### 3.5 Cardiovascular parameters

**FIGURE 5:**
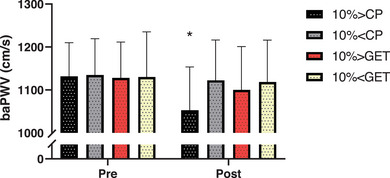
The effect of exercise intensity on brachial artery pulse wave velocity (baPWV) at baseline (Pre) and at the end of the 1 h recovery (Post). Results are displayed for each of the four criterion trials: 10% above critical power (CP), 10% below CP, 10% above the gas exchange threshold (GET) and 10% below GET. *10% > CP was significantly different from all other exercise intensities (10% < CP, 10% > GET and 10% < GET).

**FIGURE 6:**
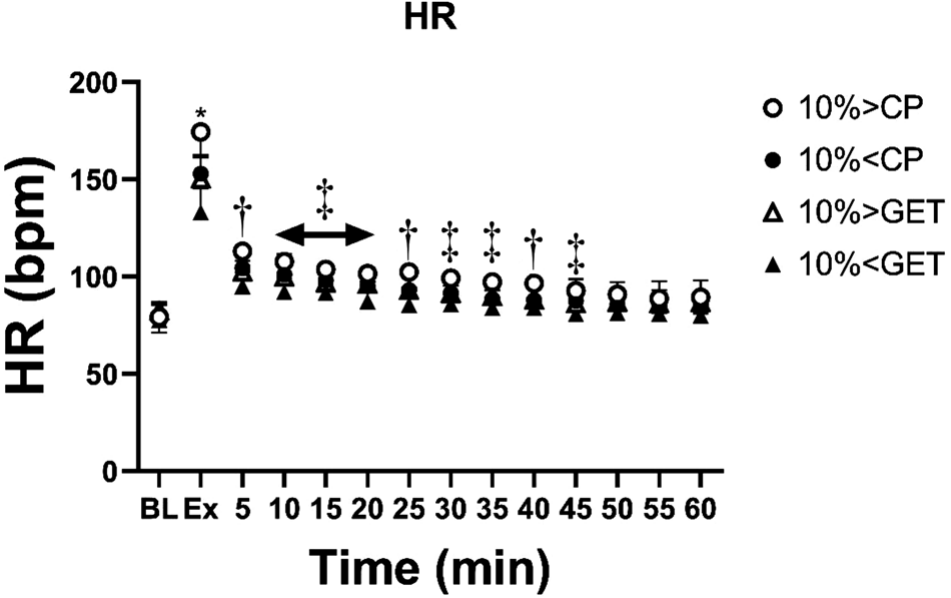
The effect of exercise intensity on heart rate (HR, group mean ± SD) at baseline, at end‐exercise, and throughout the 60 min recovery. Results are displayed for each of the four criterion trials: 10% above critical power (CP), 10% below CP, 10% above the gas exchange threshold (GET) and 10% below GET. *10% > CP was significantly different from all other exercise intensities (10% < CP, 10% > GET and 10% < GET); †10% > CP significantly different from 10% > GET and 10% < GET; ‡10% > CP significantly different from 10% < GET (*P* < 0.05).

Resting baPWV was not different across the different exercise intensities (*P* = 0.98) but was lower during recovery in the 10% > CP trial when compared to all other trials (Figure 5, *P* < 0.01). Heart rate at rest was not different across trials (*P* = 0.19), whereas during recovery from exercise 10% > CP, heart rate was greater when compared to 10% > GET and 10% < GET trials (*P* < 0.01, Figure 6).

### 3.6 Haematological parameters

Haemoglobin and haematocrit were increased by exercise in all trials (all *P* < 0.01), were lower following 60 min of recovery when compared to immediately post‐exercise, but remained elevated when compared to baseline (*P* < 0.01). These effects did not differ across different exercise intensities (*P* > 0.05). Post‐exercise changes in plasma volume were also unaffected by exercise intensity (*P* = 0.63).

## 4 DISCUSSION

The major original finding of this investigation is that the magnitude of post‐exercise hypotension was greatly increased following supra‐ compared to sub‐critical power exercise. Conversely, following exercise performed at intensities below critical power, no consistent effect of exercise intensity was observed on the magnitude of post‐exercise hypotension. These findings agree with our hypothesis and suggest that critical power represents a key threshold determining the magnitude of post‐exercise hypotension.

In the present study, we rigorously classified exercise intensity according to the intensity domain schema (Hill et al., 2002; Whipp, 1996). Specifically, subjects performing exercise below the GET displayed stable ${\dot{V}}_{O_{2}}$ across time, evidenced by the lack of difference between 3 min and end‐exercise (Figure 1). Such responses are wholly consistent with moderate intensity exercise (Whipp, 1996). During the 10% > GET and 10% < GET trials, ${\dot{V}}_{O_{2}}$ increased from 3 min to end‐exercise, indicating the presence of a slow component and consistent with exercise in the heavy domain (Jones et al., 2011; Poole et al., 1994; Whipp, 1994, 1996). Conversely, during exercise performed 10% > CP, all subjects attained ${\dot{V}}_{O_{2}\max}$ at task failure, consistent with severe‐intensity exercise (Goulding & Marwood, 2023; Goulding et al., 2021; Poole et al., 1988). Hence, using the threshold‐based exercised intensity domain schema, we were able to consistently assign subjects to their respective intensity domains, allowing the impact of exercise intensity on post‐exercise hypotension to be assessed across the full exercise intensity spectrum. Utilizing this approach, we found that the magnitude of post‐exercise hypotension was largely unaffected by exercise intensity during the trials that were performed below critical power when the total work was matched. Conversely, during recovery from exercise performed above critical power, the magnitude of post‐exercise hypotension incurred was substantially greater than that of all other trials. These findings indicate that the discrepancies between previous studies (Jones et al., 2007, 2021; MacDonald et al., 1999; Mündel et al., 2015) in this area may be explained by the observation of the present study that exercise intensity only influences the magnitude of post‐exercise hypotension when exercise is performed above critical power. Likewise, the latter observation implies that critical power is a key exercise threshold determining the magnitude of post‐exercise hypotension.

### 4.1 Comparison with previous literature

Previous work exploring the effects of exercise intensity on the magnitude of post‐exercise hypotension has yielded results conflicting with the present work. For instance, variations in exercise intensity have resulted in either similar falls in blood pressure post‐exercise (Forjaz et al., 1998; Jones et al., 2007; MacDonald et al., 1999) or greater reductions following more intense exercise (Forjaz et al., 2004; Pescatello, Franklin et al., 2004; Pescatello, Guidry et al., 2004; Piepoli et al., 1994). Subsequent to this earlier work, Jones et al. (2007) compared exercise at 40% and 75% ${\dot{V}}_{O_{2}\max}$ that was matched for total work done, and found that the magnitude of post‐exercise hypotension did not differ between the two intensities, whereas the magnitude of post‐exercise hypotension was lower following exercise performed at 40% ${\dot{V}}_{O_{2}\max}$ for a shorter duration (i.e., 30 min). These findings suggested that exercise intensity was not a key mediator of the magnitude of post‐exercise hypotension, but rather, the total work performed during an exercise bout determines the magnitude of post‐exercise hypotension. However, these previous studies examining the effect of exercise intensity on post‐exercise hypotension prescribed exercise as a percentage of ${\dot{V}}_{O_{2}\max}$ or maximal heart rate, rather than using a threshold‐based approach, as in the present study. Due to inter‐individual variation in the occurrence of the lactate threshold (approximated herein as the GET) and critical power relative to ${\dot{V}}_{O_{2}\max}$, exercise prescribed at a fixed percentage of ${\dot{V}}_{O_{2}\max}$ can result in some subjects performing exercise below their lactate threshold or critical power, and some subjects above these thresholds (Iannetta et al., 2020; Lansley et al., 2011; Meyler et al., 2023). Hence, if the goal is to determine the influence of exercise intensity on a given variable, normalizing exercise intensity as a fraction of ${\dot{V}}_{O_{2}\max}$ will result in increased inter‐individual variability in the physiological responses to exercise, which will cloud the ability of the researchers to observe the true effect of a given variable, should the effect exist. The results of the present study suggest that the findings of previous reports that exercise intensity is not a determinant of the magnitude of post‐exercise hypotension is an artefact of normalizing exercise intensity to ${\dot{V}}_{O_{2}\max}$. Moreover, these results clearly vindicate exercise intensity as a determinant of the magnitude of post‐exercise hypotension when exercise is performed above critical power.

As the purpose of our study was to compare the effects of exercise intensity alone, it was therefore necessary to match the criterion trials for the total amount of work performed. However, this raises the possibility that a longer duration of heavy‐intensity exercise would have resulted in a comparable magnitude of post‐exercise hypotension when compared to the severe‐intensity exercise bout. Conversely, the observation that the magnitude of post‐exercise hypotension following exercise above critical power was greater than typically reported in the literature for less intense, longer‐duration trials (Brito et al., 2018; Halliwill, 2001; Jones et al., 2007) strengthens the notion of the present study that critical power represents a key threshold above which the magnitude of post‐exercise hypotension is augmented.

### 4.2 Mechanisms underpinning greater magnitude of post‐exercise hypotension above critical power

Although the present study cannot resolve the mechanism responsible for the greater post‐exercise reduction in blood pressure following supra‐critical power exercise, one candidate mechanism might be useful to explore in future work. It has been demonstrated that muscular contraction increases blood flow at low exercise intensities, but has a negative impact at greater intensities (i.e., muscular contraction begins to impede limb blood flow) (Lutjemeier et al., 2005). Moreover, data from Hammer et al. (2020) have demonstrated that, during rhythmic handgrip exercise at intensities up to and including critical force (synonymous with critical power in this exercise modality), end‐exercise limb blood flow was linearly related to the constant‐force requirements of the task. However, during rhythmic handgrip exercise performed slightly above critical force, brachial artery blood flow demonstrated a plateau, being no different from the blood flow values below critical force (Hammer et al., 2020). Subsequently, during whole‐body exercise, these authors demonstrated that post‐exercise increases in limb vascular conductance and blood flow were observed following exercise above, but not below critical power (Hammer et al., 2021). These authors suggested that critical power represents an exercise intensity above which muscle contractions result in blood flow impedance, resulting in greater post‐exercise increases in skeletal muscle blood flow and limb vascular conductance (Hammer et al., 2021). This interpretation is consistent with our findings of reduced baPWV following exercise performed above critical power (i.e., indicative of greater arterial compliance, Figure 5), and with the observation that the magnitude of post‐exercise hypotension was only greater following supra‐critical power exercise. Hence, it is possible that greater blood flow impedance during supra‐critical power exercise results in greater post‐exercise increases in vascular conductance, arterial compliance and ultimately, a greater magnitude of post‐exercise hypotension. Exercise performed above critical power was shown to lead to the preferential recruitment of muscle comprising predominantly type II fibres in a rodent model (Copp et al., 2010), which might alter the production and/or release of vasoactive metabolites when compared to exercise performed below critical power. However, the factors unique to exercise performed above critical power that result in such post‐exercise alterations in limb vascular conductance, such as the profile of circulating vasoactive metabolites and/or alterations in sympathetic neural outflow (Halliwill et al., 1996), must await resolution in future research.

### 4.3 Effect of exercise intensity on core temperature

Although not the focus of the present study, we found that exercise performed above critical power resulted in a substantially greater post‐exercise hyperthermic response. This is in contrast to exercise performed below critical power, where no influence of exercise intensity on core temperature was observed during recovery. This increase in core temperature following exercise performed above critical power could potentially result in greater vascular compliance, thus partially accounting for the increased magnitude of post‐exercise hypotension (Franklin et al., 1993). Indeed, this possibility is supported by the lower baPWV response following supra‐critical power exercise, and the observation that core temperature was elevated at the same time points where mean arterial blood pressure was reduced in the present study (Figure 2). The physiological mechanism for greater post‐exercise hyperthermic responses above critical power is at present unclear and thus warrants further investigation. Specifically, special emphasis should be placed on whole‐body heat storage and heat dissipation within each exercise intensity domain to further understand this mechanism.

### 4.4 Limitations

Whilst we did recruit females for this study, all three dropped out following the critical power determination trials, and therefore the findings of this study may not be directly applicable to a female population. Indeed, whether the occurrence, magnitude and duration of post‐exercise hypotension is different between the sexes is not well‐established, and different physiological mechanisms between the sexes may be at play (Mourot et al., 2020; Queiroz et al., 2013; Rossow et al., 2010). The occurrence of metabolic thresholds relative to ${\dot{V}}_{O_{2}\max}$ has previously been shown to differ between the sexes (Ansdell et al., 2019, 2020). It is thus tempting to speculate that the discrepancies between studies regarding sex‐differences in the post‐exercise hypotensive response may be related to inappropriate normalization of exercise intensity. This suggestion should be investigated in future work. Moreover, with the measures employed in the present study, it is not possible to distinguish between mechanisms that may be responsible for the greater magnitude of post‐exercise hypotension following exercise performed above critical power. Future investigations using measures of cardiac output, limb blood flow and vascular conductance are therefore clearly warranted. The supra‐critical power trials were always conducted first, as this was necessary to match the work done in the sub‐critical power trials. Hence, this raises the possibility that our findings were related to an order effect. However, we believe that this would have been unlikely to influence our results, as participants were already familiarized to severe intensity exercise (i.e., having already performed one incremental test and five prediction trials), and the order of the subsequent criterion bouts was randomized. A further consideration is that the severe‐intensity trial was performed to task failure, whereas the heavy‐ and moderate‐intensity trials were not. It thus remains possible that the performance of work to task failure, rather than increased intensity per se, was the cause of the increased magnitude of post‐exercise hypotension following exercise above critical power. Whether this increase in magnitude of post‐exercise hypotension above versus below critical power can be attributed to the attainment of task failure represents an intriguing hypothesis to test in future research. A study with the same design as the present one but where all trials are performed to task failure could thus be used to test this hypothesis.

### 4.5 Perspectives and significance

Hypertension is a modifiable risk factor for cardiovascular disease, and exercise is a frontline therapy for reducing blood pressure (Sharman et al., 2015; Tipton, 1984). Since post‐exercise hypotension begins within the initial minutes following exercise (MacDonald, 2002) and can persist for up to 22 h in hypertensive individuals (Pescatello, Franklin et al., 2004), it has been suggested that the lowering of resting blood pressure following regular endurance training may be related to repeated instances of post‐exercise hypotension (Thompson et al., 2001). Moreover, post‐exercise hypotension has been suggested to be an important stimulus necessary for eliciting certain adaptations to chronic exercise training, such as plasma volume expansion (Halliwill, 2001; Halliwill et al., 2014). In this regard, defining the characteristics of exercise required to elicit post‐exercise hypotension, and the intensities of exercise capable of maximizing the magnitude of post‐exercise hypotension, are essential for tailoring exercise prescription to hypertensive individuals and understanding the beneficial effects of exercise on the cardiovascular system. Although the results of this study are specific to non‐hypertensive individuals, post‐exercise reductions in blood pressure are greater in hypertensive individuals, and hence the current findings may underestimate the effect of exercise intensity on post‐exercise hypotension in this population (Kenney & Seals, 1993). Hence, the results of the present study suggest that if the goal of exercise prescription is to reduce blood pressure, this may be most effectively achieved by prescribing exercise that is situated above critical power. Studies aimed at assessing the utility of supra‐ versus sub‐critical power exercise as a strategy for mitigating hypertension in affected individuals would thus appear to be a fruitful avenue for future research. Furthermore, the results of this study also suggest that individuals may be at greatest risk of developing post‐exercise syncope following exercise above critical power, which should be borne in mind when prescribing exercise to populations at risk of developing syncope (i.e., those with orthostatic intolerance).

## 5 Conclusions

We conclude that the magnitude of post‐exercise hypotension is greatly influenced by exercise intensity following exercise above, but not below, critical power. Specifically, following exercise performed at intensities that were below critical power, very little difference in the magnitude of post‐exercise hypotension was manifested. Conversely, following exercise performed above critical power, the magnitude of post‐exercise hypotension was substantially increased when compared to all other intensities when the total work performed was matched. These findings likely have profound implications for exercise prescription strategies aimed at reducing blood pressure. In particular, these results suggest that if the goal is to reduce blood pressure, one should prescribe severe‐intensity exercise situated above critical power.

## AUTHOR CONTRIBUTIONS

Tze‐Huan Lei and Richie P. Goulding, contributed to conceptualization and design. Tze‐Huan Lei, Xin Hao Liu, Hao‐Yu Li and I‐Lin Wang were responsible for data collection. Tze‐Huan Lei, Hao‐Yu Li, Yi‐Ming Chen, Xin Hao Liu, Kohei Dobashi, Yinhang Cao, Shunsaku Koga, Richie P. Goulding, Naoto Fujii, Toby Mundel, Blake Perry, Narihiko Kondo and Faming Wang were responsible for data analysis, interpretation and drafting of the article. Tze‐Huan Lei, Richie P. Goulding, Hao‐Yu Li, Narihiko Kondo, Yi‐Ming Chen, Shunsaku Koga, Kohei Dobashi, Yinhang Cao, Toby Mundel, Blake Perry, Naoto Fujii, I‐Lin Wang, Narihiko Kondo and Kohei Dobashi reviewed the article and provided critical feedback. All authors have read and approved the final version of this manuscript and agree to be accountable for all aspects of the work in ensuring that questions related to the accuracy or integrity of any part of the work are appropriately investigated and resolved. All persons designated as authors qualify for authorship, and all those who qualify for authorship are listed.

## CONFLICT OF INTEREST

There are no conflicts of interest to report. The results of this study are presented clearly, honestly, and without fabrication, falsification or inappropriate data manipulation. The results of the present study do not constitute endorsement by The Physiological Society.

## Data Availability

Data are available upon request to the authors.
